# Bone Morphogenetic Protein 7 Promotes the Differentiation of Periodontal Ligament Fibroblasts into F-Spondin-Expressing Cementoblast-like Cells During Root Canal Treatment—An In Vivo Rat Pulpectomy Model and In Vitro Human Fibroblast Study

**DOI:** 10.3390/dj13110494

**Published:** 2025-10-25

**Authors:** Hiroki Iwasawa, Yoshihiko Akashi, Kei Nakajima, Katsutoshi Kokubun, Masahiro Furusawa, Kenichi Matsuzaka

**Affiliations:** 1 Department of Endodontics, Tokyo Dental College, Tokyo 101-0061, Japan; mfurusaw@tdc.ac.jp; 2 Department of Pathology, Tokyo Dental College, Tokyo 101-0061, Japan; akashiyoshihiko@tdc.ac.jp (Y.A.); nakajimakei@tdc.ac.jp (K.N.); kkokubun@tdc.ac.jp (K.K.); matsuzak@tdc.ac.jp (K.M.)

**Keywords:** bone morphogenetic protein 7, periodontal ligament, fibroblasts, cementogenesis

## Abstract

**Background/Objectives:** The optimal healing process following root canal treatment involves biological apical sealing through new cementum formation. Bone morphogenetic protein 7 (BMP-7) has recently gained attention as a potential regulator of cementoblast differentiation and periodontal regeneration. However, its effects on periodontal ligament fibroblasts (PDLFs) and the underlying mechanisms remain incompletely understood. This study aimed to investigate whether BMP-7 induces cementoblast-like differentiation of PDLFs both in vivo and in vitro via the BMP-SMAD signaling pathway. **Methods:** In a rat pulpectomy model, root canals were treated with or without BMP-7 and examined histologically and immunohistochemically for F-spondin (Spon1) expression. In vitro, human PDLFs were stimulated with BMP-7, and analyses of mineralization, cementoblast marker expression, alkaline phosphatase activity, and SMAD-1/5/9 phosphorylation were conducted. **Results:** Immunohistochemical analysis revealed that Spon1-positive regions increased around the apical area following BMP-7 treatment, suggesting the induction of cementoblast-like differentiation. In vitro, BMP-7 enhanced the expression of cementoblast-associated genes and mineral deposition while activating SMAD-1/5/9 signaling. Phosphorylation was suppressed by the BMP receptor inhibitor LDN-193189, indicating canonical BMP-SMAD pathway involvement. **Conclusions:** Although the specific concentration range of maximal activity remains to be determined, the findings collectively suggest that BMP-7 can promote cementoblast-like differentiation of PDLFs and may contribute to apical healing through cementum-related mechanisms. These results provide mechanistic and biological insights that support the potential of BMP-7 as a modulator for biologically favorable periapical tissue regeneration following root canal therapy.

## 1. Introduction

Various morphologies of periapical tissue healing can occur after root canal treatment. For example, in cases where vital pulp remains at the apical part of the root, the healing process involves the formation of a dentin bridge. In other cases, over-filling of the root canal obturation material may lead to encapsulation of the extruded material. The ideal form of healing is considered to be the natural sealing of the root apex by newly formed cementum [1]. Calcium hydroxide agents are widely used as intracanal medications in root canal treatment, and their ability to promote hard tissue formation can result in bone-like repair at the root apex [2]. However, when the apical foramen is excessively enlarged, it is often difficult to achieve closure by cementum alone. Therefore, bioactive materials such as mineral trioxide aggregate have been proposed for physical sealing in such cases [3].

Recently, biological approaches to promote physiological cementogenesis have attracted increasing attention. Bone morphogenetic protein-7 (BMP-7), also known as osteogenic protein-1 (OP-1), is a multifunctional growth factor belonging to the transforming growth factor-β (TGF-β) superfamily. It plays key roles in organ development, tissue repair, and regeneration, and exerts anti-inflammatory effects in several systems [4]. In the craniofacial region, BMP signaling has been shown to regulate tooth morphogenesis and root development [5]. Recent studies have increasingly highlighted the cementogenic potential of BMP-7 in periodontal regeneration, demonstrating that BMP-7 delivery systems and gene-modified cells can enhance the regeneration of periodontal ligament and cementum in vivo [6,7]. Furthermore, reviews in regenerative dentistry have summarized BMP-7 as a promising candidate for biologically driven periodontal and periapical healing [8,9]. Moreover, recent evidence has indicated that cementoblasts themselves produce and secrete BMP-7, suggesting an autocrine regulatory mechanism that reinforces their own differentiation and function [10].

The periodontal ligament (PDL) is a specialized connective tissue that connects cementum to alveolar bone and contains heterogeneous cell populations, including fibroblasts, cementoblasts, osteoblasts, epithelial rests of Malassez, and mesenchymal stem cells [11]. Among these, fibroblasts are the most abundant and play essential roles in matrix remodeling and repair [12]. Therefore, the ability of PDL fibroblasts to differentiate into cementoblast-like cells is of great interest for regenerative endodontic and periodontal therapies. Cementum formation involves specific markers associated with cementoblast differentiation. F-spondin (SPON1) is an extracellular matrix glycoprotein originally identified in the neural floor plate and later found to localize preferentially along cementum and cementoblasts during root formation [13,14]. However, because SPON1 may also be expressed in fibroblastic or reparative tissues under certain conditions, it is regarded in this study as a cementoblast-associated rather than strictly cementoblast-specific marker. In addition, cementum protein-1 (CEMP1), runt-related transcription factor-2 (RUNX2), and bone gamma-carboxyglutamate (BGLAP) serve as markers of cementogenic and calcification-related differentiation.

Canonical BMP signaling acts through BMP type I receptors (ALK2, ALK3, and ALK6), which phosphorylate SMAD-1/5/9 and initiate transcriptional activation of osteo/cementogenic genes. To confirm pathway dependence, this study employs LDN-193189, a well-established inhibitor of BMP type I receptors that blocks SMAD-1/5/9 phosphorylation [15,16]. This approach enables clarification of whether BMP-7-mediated effects on PDL fibroblasts occur via the canonical BMP-SMAD signaling pathway. From a clinical standpoint, bone-like repair at the root apex can lead to ankylosis, whereas cementum-mediated regeneration maintains physiological attachment through the periodontal ligament. Therefore, promoting cementogenesis at the apical region is a biologically desirable healing outcome.

Based on these considerations, we hypothesized that BMP-7 promotes the differentiation of periodontal ligament fibroblasts into cementoblast-like cells through activation of the BMP-SMAD signaling pathway, thereby contributing to apical healing characterized by increased Spon1 expression. To test this hypothesis, in vivo experiments were performed using a rat pulpectomy model treated with or without BMP-7, while in vitro analyses were conducted using human periodontal ligament fibroblasts (HPLFs) stimulated with BMP-7 at different concentrations. The primary outcomes focused on the formation of Spon1-positive areas in the apical region and the induction of cementoblast-like markers accompanied by BMP-SMAD pathway activation in HPLFs. The purpose of this study was to investigate whether BMP-7 induces cementoblast-like differentiation of periodontal ligament fibroblasts both in vivo and in vitro via the BMP-SMAD signaling pathway, and to evaluate its potential role in promoting apical healing through cementum formation.

## 2. Materials and Methods

### 2.1. In Vivo Studies

#### 2.1.1. Animals

This study was carried out in accordance with the Guidelines for the Treatment of Experimental Animals at Tokyo Dental College (No. 230502). All in vivo experiments were conducted in compliance with the ARRIVE 2.0 guidelines. In this study, 10 nine-week-old male Wistar rats (Sankyo Lab Service, Tokyo, Japan) were used, following a similar experimental design and sample size reported in a previous study [17].

#### 2.1.2. Preparation of Root Canal Reagents

To apply reagents to the root canal of each rat, a 1% concentration of propylene glycol alginate (PGA; FUJIFILM Wako Pure Chemical Corp., Osaka, Japan) dissolved in sterile purified water was used as the control group, which was utilized as a base for Emdogain^®^ gel (Biora AB, Malmö, Sweden) in previous studies [17]. As the experimental group, recombinant human BMP-7 (ARG70032, Arigo Biolaboratories Corp., Hsinchu, Taiwan) was added to 1% PGA to reach a concentration of 50 µg/mL, based on previous reports indicating that the optimal total dose of BMP-7 for bone regeneration in vivo is approximately 50 µg, as demonstrated in a rat segmental bone defect model [18]. This nominal concentration was selected to compensate for potential losses due to adsorption, diffusion, and inactivation within the root canal gel.

#### 2.1.3. Experimental Procedure

**Figure 1:**
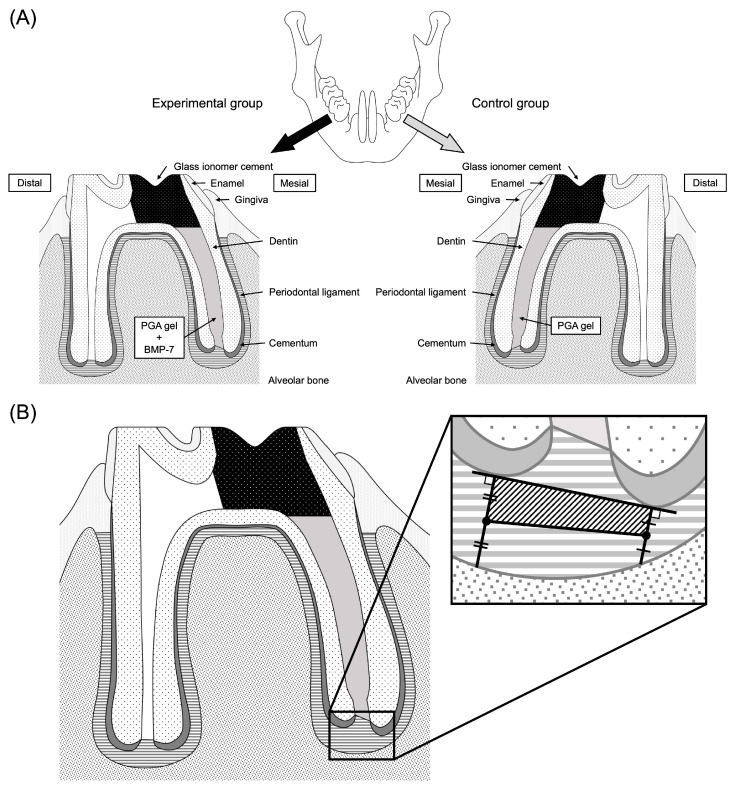
Scheme of the in vivo study of rat mandibular first molars. (**A**) The pulp of the mesial root canal was removed and the root canal was prepared with #10–#20/.04 Ni-Ti rotary files. After cleaning and drying, the left side was filled with PGA and the right side was filled with PGA containing BMP-7. The access cavities in the crown were sealed with glass ionomer cement. (**B**) The area of interest at the root apex was defined as a square and the immunopositive area was calculated.

Each rat was anesthetized with isoflurane (FUJIFILM Wako Pure Chemical Corp.) and a triple anesthetic mixture of medetomidine (Domitor; Nippon Zenyaku Kogyo Co., Ltd., Fukushima, Japan), midazolam (Midazolam Sandoz; Sandoz, Yamagata, Japan) and butorphanol (Vetorphale; Meiji Seika Pharma Co., Ltd., Tokyo, Japan). After local anesthesia with lidocaine (Epilido cartridge; Nipro Co., Osaka, Japan), a rubber-dam isolation technique was performed using a custom-made rubber-dam clamp for rats (YDM Co., Tokyo, Japan) and a rubber-dam sheet. In addition, a caulking material (Dentto-Dam; Mediclus Co., Ltd., Cheongju, Republic of Korea) was applied to the gaps between the clamps and sheets to accomplish complete isolation. A cavity was then prepared on the occlusal surface of the mandibular first molar of each rat using a 0.5 mm carbide bur (Shofu carbide round bur No. 1/4; Shofu, Kyoto, Japan) and the coronal pulp was removed using a micro-excavator (OK Micro-exca; Seto, Ibaraki, Japan) under a surgical loupe (Admetec Solutions Ltd., Haifa, Israel) and a stereoscopic microscope (Stemi 508; Carl Zeiss, Oberkochen, Germany). The root canal length of the mesial root was measured using an electrical root canal length measuring device (Root ZX; J. Morita Corp., Tokyo, Japan) and the working length was set at 3.5 mm, which is the root canal length (4 mm) - 0.5 mm. The mesial root canal was enlarged with a nickel-titanium rotary file (ProTaper Gold SX; Dentsply Sirona, Tokyo, Japan) in the coronal root canal. After penetrating the root canal with a #8 K file, an apical root canal was prepared with 0.04 taper nickel-titanium rotary files (Race; FKG, La Chaux-de-Fonds, Switzerland) from #10 to #20 at the working length. Each root canal was irrigated with 10% sodium hypochlorite (Neo Cleaner “Sekine”; Neo Dental Chemical Products, Tokyo, Japan) and 17% ethylenediaminetetraacetic acid (EDTA; Ci Medical Co., Ltd., Ishikawa, Japan) by passive ultrasonic irrigation (#20 U-file; Zipperer Co., München, Germany). The concentration of 10% sodium hypochlorite was selected to reproduce the clinical conditions used in our college hospital, where this concentration is routinely employed for endodontic irrigation. After the root canal was rinsed with purified water and wiped with absorbent paper, the root canal on the left side was filled with 1% PGA as the control group, and the root canal on the right side was filled with 1% PGA containing 50 μg/mL BMP-7 as the experimental group. The coronal access cavities were then sealed with a light-curing glass ionomer cement (Ionosit-Baseliner; DMG, Chemisch-Pharmazeutische Fabrik GmbH, Hamburg, Germany). The occlusal surfaces were then adjusted to avoid occlusion with the opposing teeth (Figure 1A).

#### 2.1.4. Histological Observations

Each rat was sacrificed 14 or 28 days after treatment by an overdose of anesthetic. The mandibular first molars of each rat were removed with the mandibular bone. The excised mandibular bone was immersed and fixed in 4% paraformaldehyde at room temperature for 1 day and then demineralized with 10% EDTA at 4 °C for 28 days. The EDTA was changed every 3 days. After demineralization, the tissues were embedded in paraffin and then cut into 5 μm parasagittal sections using a sliding microtome. The sections were stained with hematoxylin and eosin (H-E) for microscopic observations (*n* = 5) using a light microscope (Olympus BX51; Olympus Corp., Tokyo, Japan) equipped with imaging software (cellSens Standard version 1.18; Olympus Corp.).

#### 2.1.5. Immunohistochemical Observations

Paraffin sections of specimens at days 14 and 28 after treatment were used for immunohistochemical observations. The paraffin sections were deparaffinized with xylol and were antigen-activated for 20 min at 37 °C with a Trypsin Antigen Retrieval Kit (ab259256, Abcam, Cambridge, MA, USA). Each section was immersed in methanol containing 0.3% hydrogen peroxide solution at room temperature to block endogenous peroxidase activity. The sections were blocked with 10% goat serum for 30 min at room temperature to reduce non-specific binding. Sections were then reacted with the primary antibody at 4 °C overnight. The primary antibody used was a rabbit anti-F-spondin antibody (Spon1; 1:100, ab215165, Abcam) as a cementoblast marker. The primary antibody was replaced with 1% goat serum as a negative control. The sections were then reacted with Mach 2 rabbit HRP-polymer secondary antibody (BRR522G, Biocare Medical, Concord, CA, USA) for 30 min at room temperature, after which the sections were stained with DAB and nuclei were stained with hematoxylin.

#### 2.1.6. Measurement of Positively Immunostained Structures and Statistical Analysis

The immunostained images obtained were analyzed using Fiji software (ImageJ2, Version: 2.14.0) [19]. For analysis, a tangent line was drawn at the tip of the mesial root apex and a vertical line was drawn from that tangent line to the alveolar bone. The area of a square connecting the midpoints of those perpendicular lines was set as the area of interest (Figure 1B), because F-spondin has been reported to be predominantly localized in the periodontal ligament adjacent to the root surface rather than the alveolar bone. The periodontal ligament area and the Spon1-positive area of the region of interest were measured and the ratio of the Spon1-positive area was calculated as: Spon1-positive area/Area of interest × 100 (%) (*n* = 5). All image analyses were performed by a single calibrated examiner using a consistent evaluation protocol to ensure reproducibility.

### 2.2. In Vitro Studies

#### 2.2.1. Cells

Human periodontal ligament fibroblasts (HPLFs) were purchased from ScienCell Research Laboratories (#2630, San Diego, CA, USA). HPLFs were incubated at 37 °C in 95% air mixed with 5% CO_2_ and HPLFs at passage 9 were used in this study, as commercially available cells derived from a single healthy donor to ensure standardization. To minimize biological variability and maintain consistency across all experiments, cells at the same passage number (passage 9) were used throughout the study.

#### 2.2.2. Cell Culture Medium

**Figure 2:**
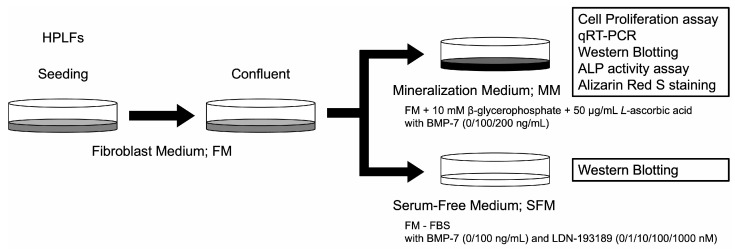
Overview of HPLFs used in the in vitro studies. HPLFs were cultured in FM. MM was used for experiments with BMP-7 at concentrations of 0, 100 or 200 ng/mL. SFM was used for experiments with BMP-7 at 0 or 100 ng/mL and LDN-193189 at 0, 1, 10, 100 or 1000 nM. FM; fibroblast medium, MM; mineralization medium, SFM; serum-free medium.

HPLFs were cultured in 100 mm tissue culture dishes (Corning, Bedford, MA, USA) with Fibroblast Medium (FM, ScienCell, #2301) containing 2% fetal bovine serum (FBS, #0010), fibroblast growth supplement (FGS, #2352) and 1% penicillin/streptomycin solution (P/S, #0503). Mineralization medium (MM) was prepared by adding 10 mM β-glycerophosphate and 50 μg/mL L-ascorbic acid to the FM. Serum-free medium (SFM) was prepared by excluding FBS from the FM. Recombinant human BMP-7 was dissolved in sterile water and added to MM at 100 or 200 ng/mL and to SFM at 100 ng/mL. BMP-7 was not added to the medium in the control group. Various media were used for each experiment (Figure 2).

#### 2.2.3. Cell Proliferation Assay

The WST-8 assay (Cell Counting Kit-8; Dojindo, Kumamoto, Japan), which is based on the cleavage of the tetrazolium salt WST-8 to formazan by cellular mitochondrial dehydrogenase, was used to study cell proliferation by measuring formazan in the mitochondria of cells. HPLFs were seeded in 24-well plates at a density of 2.5 × 10^3^ cells/cm^2^ in three different media: MM + BMP-7 (0, 100 or 200 ng/mL). After 1, 3 and 5 days of culture, 50 μL Cell Counting Kit-8 reagent was added to each well and was incubated at 37 °C, 5% CO_2_ for 1 h. The absorbance at 450 nm was measured using a microplate reader (Synergy H1; Bio Tek Gen5, Bio Tek, Winooski, VT, USA). The media were changed every 3 days (*n* = 4, and each sample was measured in duplicate).

#### 2.2.4. BMP Signaling Pathway

The signaling pathway by which BMP-7 affects HPLFs was investigated. HPLFs were seeded in 60 mm dishes at a density of 1.0 × 10^5^ cells/cm^2^. After reaching confluence, HPLFs were washed twice with PBS and the medium was replaced with SFM and the HPLFs were starved. After three hours, the medium was changed to SFM supplemented with 0 or 100 ng/mL BMP-7 and 0, 1, 10, 100 or 1000 nM LDN-193189 (ab278073, Abcam), a BMP receptor inhibitor. After 24 h, the HPLFs were collected and evaluated by Western blot analysis. Western blot data were qualitatively evaluated. The band intensities of the Western blot images were quantified by densitometric analysis using ImageJ software (version 1.54g; National Institutes of Health, Bethesda, MD, USA). The detailed densitometric data are presented in the Supplementary Materials (Table S1).

#### 2.2.5. Quantitative Reverse Transcription Polymerase Chain Reaction (qRT-PCR) Assay

HPLFs were seeded in 6-well plates at a density of 1 × 10^4^ cells/cm^2^ and were cultured in FM. Three days after seeding, when the cells were confluent, was defined as Day 0 and the medium was replaced with MM + BMP-7 (0, 100 or 200 ng/mL). HPLFs were collected on days 7 or 14 from Day 0. Total RNAs were extracted using a RNeasy Mini Kit (Qiagen, Hilden, Germany) after which complementary DNAs (cDNAs) were reverse transcribed using ReverTra Ace qPCR RT Master Mix with gDNA Remover (Toyobo Co., Osaka, Japan). Quantitative reverse transcription polymerase chain reaction (qRT-PCR) analyses were performed using TaqMan Gene Expression Assays (Applied Biosystems, Waltham, MA, USA). In this study, we used F-spondin (*SPON1*) mRNA (Hs01120488_m1) as a cementoblast differentiation marker. In addition, cementum protein 1 (*CEMP1*) mRNA (Hs04185363_s1) was used as a cementoblast-related marker, runt-related transcription factor 2 (*RUNX2*) mRNA (Hs01047973_m1) and bone gamma-carboxyglutamate protein (*BGLAP*) mRNA (Hs01587814_g1) were used as markers of calcification. Glyceraldehyde-3-phosphate dehydrogenase (*GAPDH*) mRNA (Hs03929097_g1) was used as a reference gene. qRT-PCR was performed using a 7500 Fast Real-Time PCR System (Applied Biosystems). The relative expression levels of genes of interest were estimated using the ΔΔ threshold cycle (ΔΔCt) method. The mRNA expression levels were corrected based on *GAPDH* mRNA expression levels, and target gene expression levels were subjected to relative quantitative analysis (*n* = 4, and each sample was measured in duplicate).

#### 2.2.6. Western Blot Analysis

HPLFs were seeded in 60 mm dishes at a density of 2 × 10^4^ cells/cm^2^ and were cultured in FM. Three days after the cells became confluent was designated as Day 0, and the medium was replaced with MM + BMP-7 (0, 100 or 200 ng/mL) and refreshed every 3 days. After incubation for 21 days, the HPLFs were lysed in Pierce IP Lysis Buffer (Thermo Fisher Scientific, Waltham, MA, USA) with a protease and phosphatase cocktail and lysates were centrifuged at 12,000 rpm for 10 min at 4 °C. The cell lysates were collected, and total protein levels were measured using the BCA protein assay. Equal amounts of proteins from each sample were loaded per lane and were separated using 10% sodium dodecyl sulfate-polyacrylamide gel electrophoresis (SDS-PAGE) gels (Bio-Rad Laboratories, Inc., Hercules, CA, USA) after which they were transferred to polyvinylidene fluoride (PVDF) membranes (iBlot2 Transfer Stack) using an iBlot2 Dry Blotting system (Invitrogen, Carlsbad, CA, USA). Each membrane was blocked for 1 h using Blocking One (Nacalai Tesque, Kyoto, Japan) and was then incubated overnight at 4 °C with the following primary antibodies: SPON1 (1:1000, #40372, Signalway Antibody LLC, College Park, MD, USA), CEMP1 (1:500, ab169514, Abcam) and GAPDH (1:10,000, ab8245, Abcam). For the BMP signaling pathway, antibodies for total SMAD-1/5/9 (1:1000, ab80255, Abcam), phospho-SMAD-1/5/9 (1:1000, #13820, Cell Signaling Technology, Beverly, MA, USA), and GAPDH (1:10,000) were used. The membranes were washed with TBS containing Tween-20 (TBS-t) for 30 min and were then incubated with the corresponding horseradish peroxidase-conjugated secondary antibodies (1:3000, #170-6515; 1:10,000, #170-6516, Bio-Rad Laboratories, Inc.) for 1 h at room temperature. After the membranes were washed with TBS-t for 30 min, bound antibodies were detected using an ECL Prime kit (RPN2232, Amersham, GE Healthcare Bioscience, Piscataway, NJ, USA) and an ImageQuant LAS 4000 mini (GE Healthcare Bioscience). Western blot data were qualitatively evaluated. The band intensities of the Western blot images were quantified by densitometric analysis using ImageJ software (version 1.54g; National Institutes of Health, Bethesda, MD, USA). The detailed densitometric data are presented in the Supplementary Materials (Table S2).

#### 2.2.7. Alkaline Phosphatase (ALP) Activity Assay

HPLFs were seeded in 6-well plates at a density of 1 × 10^4^ cells/cm^2^ and were cultured in FM. Three days after the cells became confluent was designated as Day 0, and the medium was replaced with MM + BMP-7 (0, 100 or 200 ng/mL) and refreshed every 3 days. After incubation for 21 days, the cells were lysed in Pierce IP Lysis Buffer (Thermo Fisher Scientific) with a protease and phosphatase cocktail and lysates were centrifuged at 12,000 rpm for 10 min at 4 °C. The supernatants were analyzed using a LabAssay ALP kit (FUJIFILM Wako Pure Chemical Corp.), according to the manufacturer’s instructions. Total protein levels were quantified using a BCA assay kit (Pierce). ALP production was normalized to the total protein amount (*n* = 6, and each sample was measured in duplicate).

#### 2.2.8. Alizarin Red S (ARS) Staining

HPLFs were seeded in 12-well plates at a density of 1 × 10^4^ cells/cm^2^ and were cultured in FM. Three days after the cells became confluent was designated as Day 0, and the medium was replaced with MM + BMP-7 (0, 100 or 200 ng/mL) and refreshed every 3 days. After incubation for 28 days, the cells were washed with PBS and fixed with 2% paraformaldehyde (Nacalai Tesque) for 10 min at room temperature. After fixation, each well was washed twice with purified water and was stained with ARS staining solution (pH 6.4) (Sigma-Aldrich, St. Louis, MO, USA) for 10 min at 37 °C. After staining, the ARS staining solution was aspirated from the wells, and the wells were washed with purified water 3 times and then air dried. Cells were imaged using a single-lens reflex camera (Canon EOS Kiss X7i; Canon Inc., Tokyo, Japan). The presence of calcified deposits was visually assessed, and stained regions were identified in Fiji software by applying a consistent threshold value across all images. Areas exceeding this threshold were regarded as positively stained, and the stained area ratio per well was then calculated (*n* = 12). All image analyses were performed by a single calibrated examiner to ensure consistency.

#### 2.2.9. Statistical Analysis

Quantitative data are expressed as means ± standard deviation (SD) using EZR software version 1.61 (Saitama Medical Center, Jichi Medical University, Saitama, Japan) [20], which is a graphical user interface for R (The R Foundation for Statistical Computing, Vienna, Austria, version 4.2.2). The normality of the data distribution was first examined using the Kolmogorov–Smirnov test. Subsequently, evaluation of quantitative data was analyzed by relative evaluation using one-way ANOVA and two-way ANOVA analysis with post hoc Tukey’s multiple comparison test. In the in vivo experiment, one periapical tissue surrounding a single root apex was regarded as a single analytical unit, and data from both sides of each animal were treated independently. In the in vitro experiments, one cell population derived from a single culture well was considered as one analytical unit. Statistical significance was set at *p* < 0.05 or *p* < 0.01.

## 3. Results

### 3.1. In Vivo Studies

#### 3.1.1. Histological Observations

**Figure 3:**
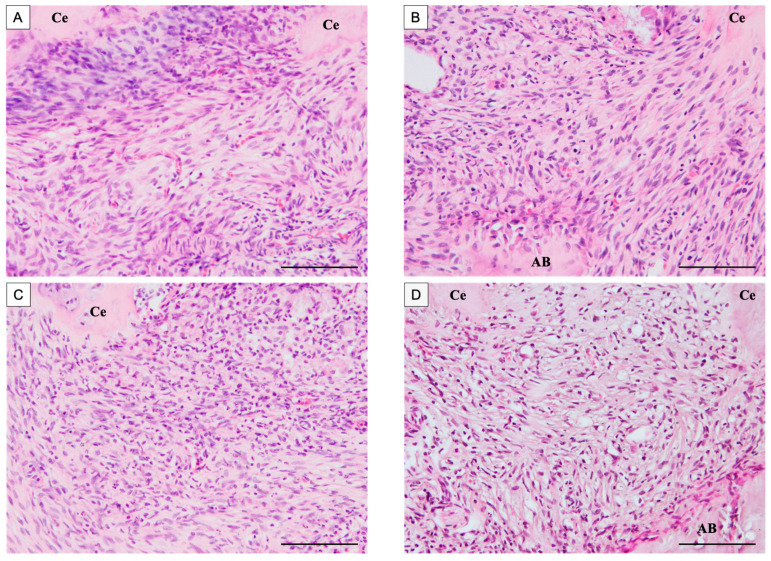
H-E stained images of the apical area of the mesial root after root canal treatment. The control group was filled with PGA only, and the experimental group was filled with PGA and 50 µg/mL BMP-7. (**A**) Control group at 14 days postoperatively. (**B**) BMP-7 group at 14 days postoperatively. (**C**) Control group at 28 days postoperatively. (**D**) BMP-7 group at 28 days postoperatively. (*n* = 5) In all groups, the periodontal ligament at the root apex was mainly composed of short spindle-shaped cells, and no continuous formation of cementum along the root surface was observed. Scale bars = 100 μm. Ce: cementum, AB: alveolar bone.

The mesial root apex of each rat mandibular first molar was observed by H-E staining. In all groups, the periodontal ligament tissue of the root apex was predominantly occupied by short spindle cells. No continuous formation of cementum along the root surface was observed in any group (Figure 3A–D).

#### 3.1.2. Immunohistochemical Observations

**Figure 4:**
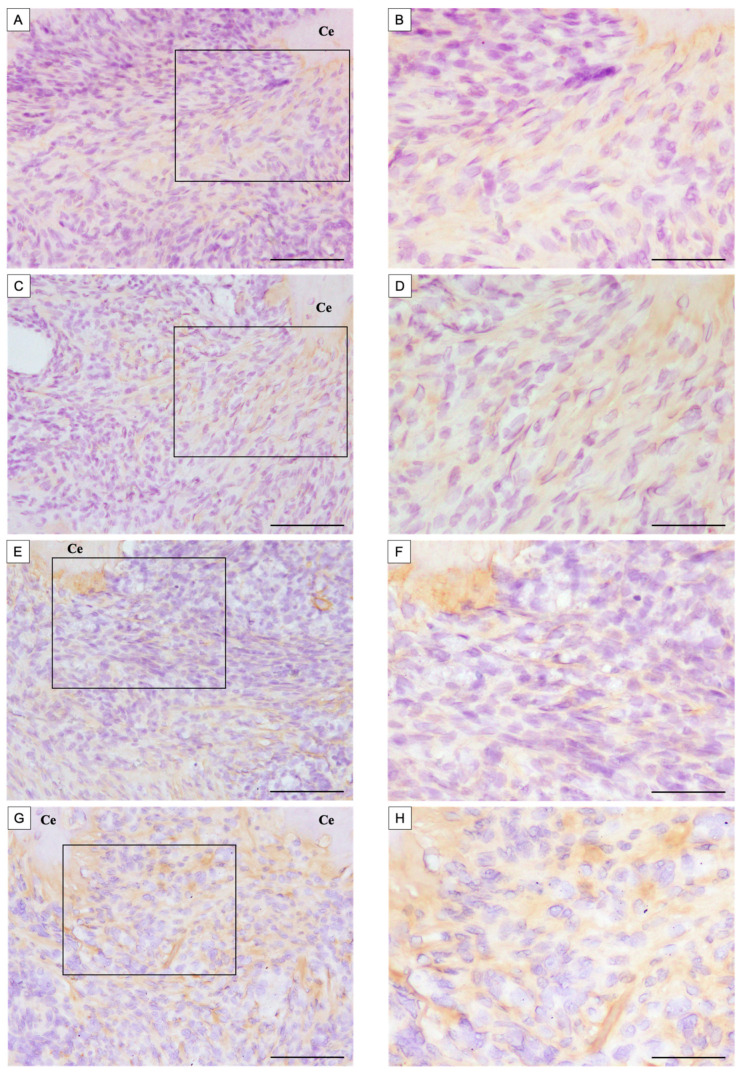

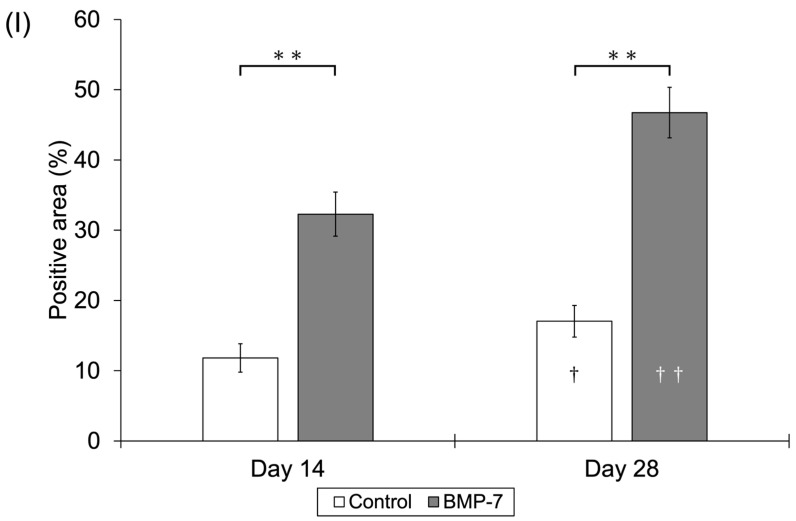
Immunohistochemical staining of Spon1 of the apical area of the mesial root after root canal treatment. The control group was filled with PGA only, and the experimental group was filled with PGA and 50 µg/mL BMP-7. (**A**,**B**) Control group at 14 days postoperatively. (**C**,**D**) BMP-7 group at 14 days postoperatively. (**E**,**F**) Control group at 28 days postoperatively. (**G**,**H**) BMP-7 group at 28 days postoperatively. (**B**,**D**,**F**,**H**) Expanded images of the boxed areas indicated in (**A**,**C**,**E**,**G**), respectively. (*n* = 5) (**A**) A few Spon1-positive areas were observed. (**B**) Spon1-positive areas were observed compared to the control group. (**C**) Spon1-positive areas were observed compared to Day 14. (**D**) The most Spon1-positive areas were observed compared to other groups. Scale bars = 100 μm; magnified image Bars = 50 μm. Ce: cementum, AB: alveolar bone. (**I**) The ratio of Spon1-positive area in the apical periodontal ligament calculated as: (Spon1-positive area/Area of interest) × 100%. Asterisks indicate significant differences among groups on the same postoperative day (**: *p* < 0.01), whereas daggers indicate significant differences between Day 14 and Day 28 within the same group (†: *p* < 0.05, ††: *p* < 0.01).

Spon1-positive reactions were visible around the root cementum in all groups. At 14 days after root canal treatment, a few Spon1-positive areas were observed at the root apex in the control group (Figure 4A,B). More Spon1-positive areas were observed in the BMP-7-treated experimental group at 14 days compared to the control group (Figure 4C,D). On the other hand, at 28 days after root canal treatment, the control group showed a greater extent of Spon1-positive areas than at day 14 (Figure 4E,F). Most Spon1-positive areas were observed in the root apex in the BMP-7-treated experimental group at 28 days compared to the control group (Figure 4G,H). The ratio of the Spon1-positive area to the area of interest was 11.8 ± 2.02% for the control group and 32.3 ± 3.14% for the BMP-7-treated experimental group on postoperative day 14, and 17.0 ± 2.25% for the control group and 46.7 ± 3.60% for the BMP-7-treated experimental group on postoperative day 28 (Figure 4I). Spon1 positivity was significantly higher in the BMP-7-treated experimental group compared to the control group on both postoperative days 14 and 28. Furthermore, Spon1 positivity was significantly increased on day 28 compared to day 14 in both groups.

### 3.2. In Vitro Studies

#### 3.2.1. Cell Proliferation Assay

**Figure 5:**
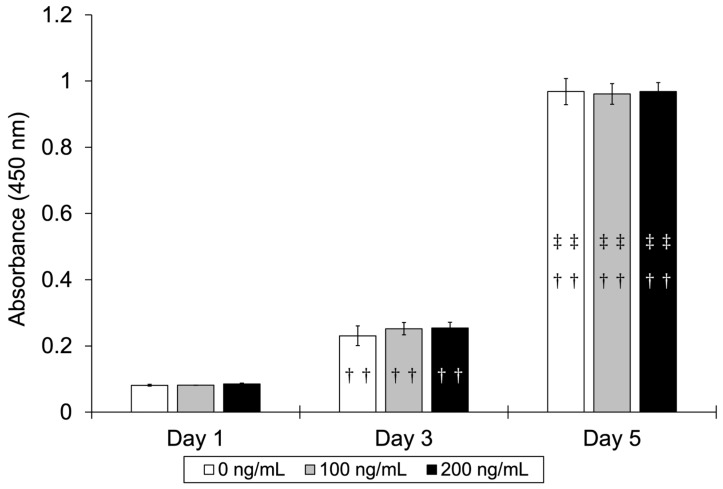
Cell proliferation rate of HPLFs cultured with different BMP-7 concentrations. Significant increases in cell proliferation were observed in all groups from Day 1 to Day 5, but no significant differences were found among the concentrations. (*n* = 4) Daggers indicate significant differences compared with Day 1, and double daggers indicate significant differences compared with Day 3 (††: *p* < 0.01 vs. Day 1; ‡‡: *p* < 0.01 vs. Day 3).

The absorbance values (mean ± SD) for the control group (0 ng/mL) were 0.08 ± 0.00 on day 1, 0.23 ± 0.03 on day 3, and 0.97 ± 0.04 on day 5. For the 100 ng/mL BMP-7 group, the values were 0.08 ± 0.00, 0.25 ± 0.02, and 0.96 ± 0.03, respectively, while for the 200 ng/mL BMP-7 group, they were 0.09 ± 0.00, 0.25 ± 0.02, and 0.97 ± 0.03, respectively. Although HPLFs on days 3 and 5 showed significantly higher cell proliferation rates than on day 1 in all groups, there were no significant differences among the three groups at any time point (Figure 5).

#### 3.2.2. BMP Signaling Pathway

**Figure 6:**
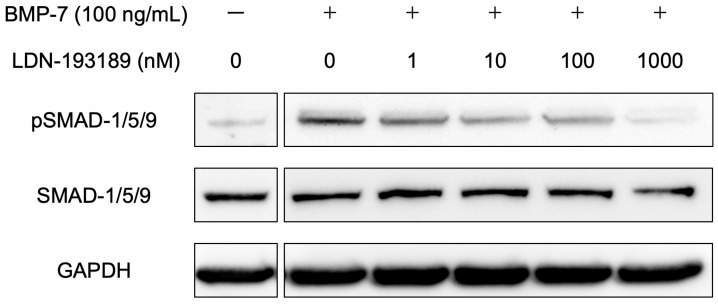
Protein expression levels in the BMP signaling pathway of HPLFs and the inhibition of BMP. SMAD-1/5/9 and phospho-SMAD-1/5/9 were qualitatively detected by Western blot analysis. Stimulation of HPLFs with 100 ng/mL BMP-7 resulted in the phosphorylation of SMAD-1/5/9, but the addition of LDN-193189, an inhibitor of BMP signaling, qualitatively decreased the band intensity of phospho-SMAD-1/5/9 as its concentration increased. Representative qualitative results are shown.

The expression of SMAD-1/5/9 was qualitatively detected in all groups (Figure 6). The expression of phospho-SMAD-1/5/9 was qualitatively observed after treatment with 100 ng/mL BMP-7, but its band intensity decreased as the concentration of the BMP-7 inhibitor LDN-193189 increased. These findings were based on qualitative evaluation of band intensity rather than quantitative densitometric analysis. Additional data, including the densitometric analysis (Table S1) and uncropped Western blot images (Figure S1), are provided in the Supplementary Materials (File S1).

#### 3.2.3. qRT-PCR Assay

**Figure 7:**
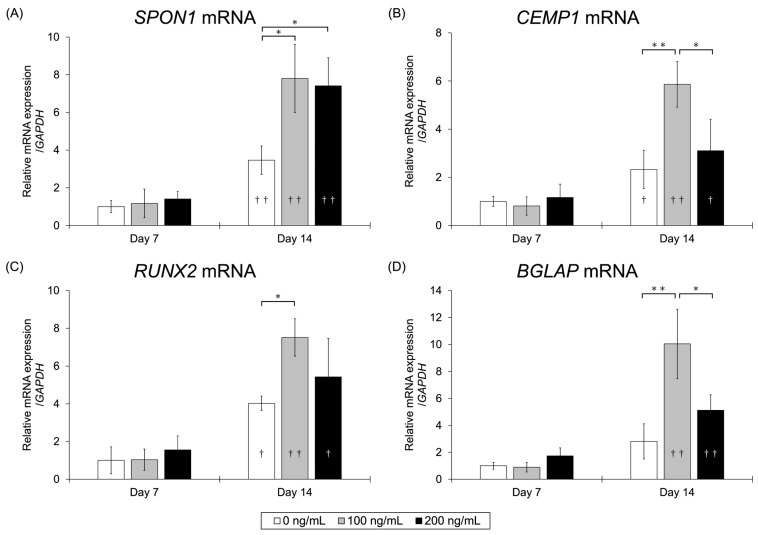
mRNA expression levels at each BMP-7 concentration assessed by qRT-PCR analysis. The expression levels of each mRNA were higher in the 100 ng/mL BMP-7-treated group compared to the control group. (**A**) *SPON1* mRNA expression levels. (**B**) *CEMP1* mRNA expression levels. (**C**) *RUNX2* mRNA expression levels. (**D**) *BGLAP* mRNA expression levels. (*n* = 4) Asterisks indicate significant differences among BMP-7 concentrations on the same day (*: *p* < 0.05, **: *p* < 0.01), whereas daggers indicate significant differences between Day 7 and Day 14 within the same BMP-7 concentration (†: *p* < 0.05, ††: *p* < 0.01).

The relative expression levels (mean ± SD) of *SPON1* on Day 7 were 1.00 ± 0.32, 1.17 ± 0.76, and 1.41 ± 0.39 for the 0, 100, and 200 ng/mL BMP-7 groups, and on Day 14 were 3.47 ± 0.75, 7.80 ± 1.81, and 7.41 ± 1.48, respectively. The mRNA expression level of *SPON1* was significantly higher in the groups treated with 100 or 200 ng/mL BMP-7 compared to the control group on Day 14 (*p* < 0.05) (Figure 7A). In all groups, *SPON1* expression on Day 14 was higher than that on Day 7. The relative expression levels of *CEMP1* on Day 7 were 1.00 ± 0.20, 0.81 ± 0.38, and 1.16 ± 0.55, and on Day 14 were 2.33 ± 0.80, 5.86 ± 0.95, and 3.10 ± 1.30 for the 0, 100, and 200 ng/mL BMP-7 groups, respectively. The mRNA expression level of *CEMP1* was significantly higher in the 100 ng/mL BMP-7-treated group compared to the control group (*p* < 0.01) and in the 200 ng/mL BMP-7-treated group (*p* < 0.05) on Day 14 (Figure 7B). The relative expression levels of *RUNX2* on Day 7 were 1.00 ± 0.71, 1.03 ± 0.57, and 1.56 ± 0.74, and on Day 14 were 4.02 ± 0.39, 7.51 ± 0.99, and 5.43 ± 2.04 for the 0, 100, and 200 ng/mL BMP-7 groups, respectively. The mRNA expression level of *RUNX2* was significantly higher in the 100 ng/mL BMP-7-treated group compared to the control group (*p* < 0.05) on Day 14 (Figure 7C). The relative expression levels of *BGLAP* on Day 7 were 1.00 ± 0.26, 0.89 ± 0.35, and 1.73 ± 0.60, and on Day 14 were 2.81 ± 1.31, 10.04 ± 2.58, and 5.12 ± 1.15 for the 0, 100, and 200 ng/mL BMP-7 groups, respectively. The mRNA expression level of *BGLAP* was significantly higher in the 100 ng/mL BMP-7-treated group compared to the control group (*p* < 0.01) and the 200 ng/mL BMP-7-treated group (*p* < 0.05) on Day 14 (Figure 7D). In all groups, the expression levels of these mRNAs were higher on Day 14 than on Day 7 (Figure 7A–D).

#### 3.2.4. Western Blot Analysis

**Figure 8:**
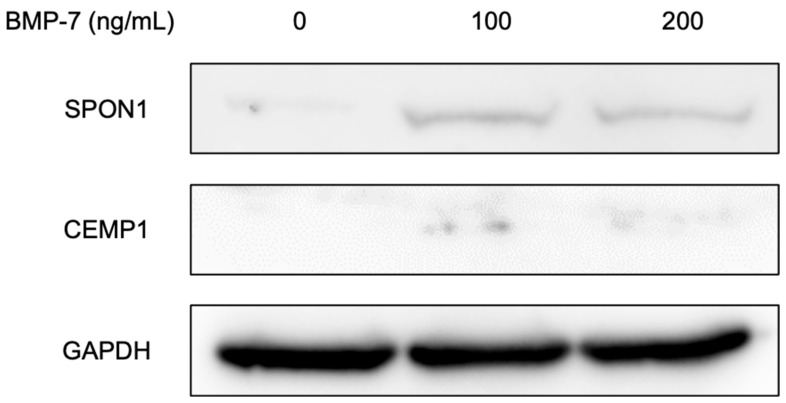
Detection of proteins in HPLFs following treatment at each BMP-7 concentration by Western blot analysis. SPON1 and CEMP1 proteins were qualitatively detected, showing the strongest bands in the 100 ng/mL BMP-7-treated group, followed by the 200 ng/mL BMP-7-treated group, whereas minimal or no expression was observed in the control. Lane 1, 0 ng/mL BMP-7-treated group as a control; lane 2, 100 ng/mL BMP-7-treated group; lane 3, 200 ng/mL BMP-7-treated group. Representative qualitative results are shown.

The SPON1 protein was qualitatively detected with the strongest expression in the 100 ng/mL BMP-7-treated group, followed by the 200 ng/mL BMP-7-treated group, whereas its expression in the control group was minimal (Figure 8). Similarly, CEMP1 protein expression was qualitatively observed in the 100 ng/mL BMP-7-treated group and only slightly in the 200 ng/mL BMP-7-treated group, but was not detected in the control. These findings were based on qualitative evaluation of band intensity rather than quantitative densitometric analysis. Additional data, including the densitometric analysis (Table S2) and uncropped Western blot images (Figure S2), are provided in the Supplementary Materials (File S1).

#### 3.2.5. ALP Activity Assay

**Figure 9:**
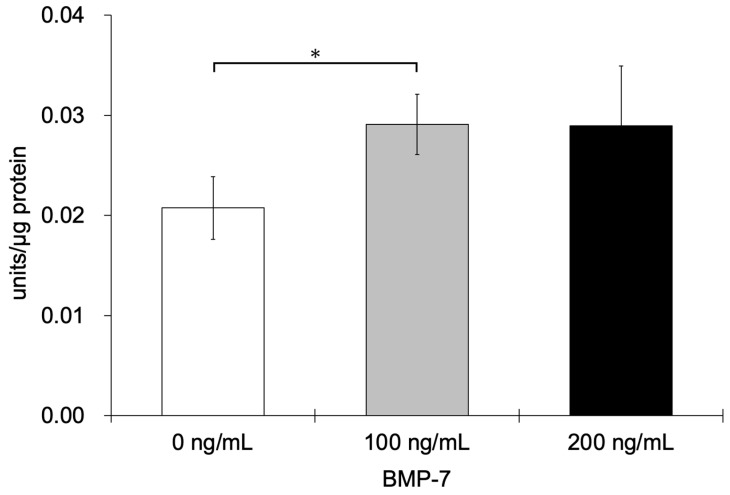
ALP activity of HPLFs following treatment at each BMP-7 concentration. ALP activity was significantly higher in the 100 ng/mL BMP-7-treated group compared to the control group (*n* = 6) (*: *p* < 0.05).

The ALP activity of HPLFs was 0.02 ± 0.00, 0.03 ± 0.00, and 0.03 ± 0.01 for the 0, 100, and 200 ng/mL BMP-7-treated groups, respectively. The ALP activity of HPLFs was significantly higher in the 100 ng/mL BMP-7-treated group compared to the control group (*p* < 0.05) (Figure 9).

#### 3.2.6. ARS Staining

**Figure 10:**
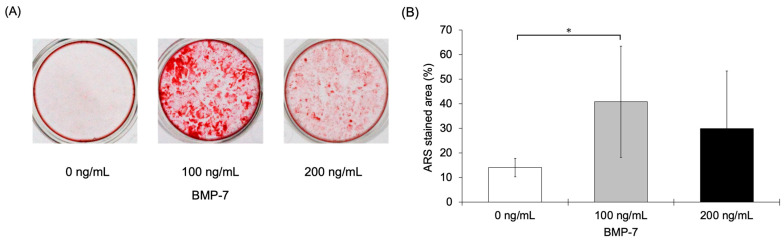
ARS staining assay of HPLFs following treatment at each BMP-7 concentration. (**A**) Representative images showing qualitative evaluation of calcium deposition by ARS staining. (**B**) The stained area was measured to semi-quantitatively assess mineralization, and calcification was significantly higher in the 100 ng/mL BMP-7-treated group compared to the control group (*n* = 12) (*: *p* < 0.05).

After 28 days of incubation of HPLFs in MM, calcium deposition was qualitatively observed to increase in the BMP-7-treated groups (Figure 10A). To semi-quantitatively evaluate mineralization, the stained area was measured. The calcified area (mean ± SD) was 14.09 ± 3.78, 40.84 ± 22.65, and 29.89 ± 23.43 for the 0, 100, and 200 ng/mL BMP-7-treated groups, respectively. In particular, the area of calcified nodules was significantly increased in the 100 ng/mL BMP-7-treated group compared to the control group (*p* < 0.05) (Figure 10B).

## 4. Discussion

In the in vivo study, immunohistochemical staining revealed the presence of Spon1-positive areas around the root apex in the BMP-7-treated experimental group. Although no distinct cementum-like hard tissue was detected by H-E staining, the appearance of Spon1-positive regions suggests that BMP-7 may have induced cementoblast-like differentiation in cells located within the apical periodontal ligament. Spon1 is an extracellular matrix glycoprotein known to participate in tissue organization and has been identified as a marker associated with cementoblast differentiation [10,14]. The localization of Spon1 around the apical region after BMP-7 treatment implies that this growth factor may activate cementogenic mechanisms within the periodontal ligament space. From a clinical standpoint, cementum-mediated sealing of the root apex represents the most favorable healing pattern following root canal therapy, as it restores the physiological periodontal ligament attachment and minimizes the risk of ankylosis. Histologically, bone-like repair within the apical region has often been associated with the loss of the periodontal ligament space; therefore, a healing process involving cementoblast-like differentiation is regarded as a more biologically appropriate response. These findings suggest that BMP-7 can modulate the apical microenvironment in a manner that promotes physiological tissue regeneration rather than pathological bone replacement [21,22].

BMP-7, a member of the TGF-β superfamily, is well recognized for its osteogenic and cementogenic potential. Previous studies have shown that BMP-7 promotes the differentiation of cementoblasts and periodontal ligament-derived stem cells, leading to mineralized matrix formation [21,22]. In the present in vivo experiment, the upregulation of Spon1 in the BMP-7-treated roots supports this concept and indicates that BMP-7 promotes cementoblast-like activity even in a post-developmental healing environment. Furthermore, the increased Spon1 expression in the absence of extensive inflammation suggests that BMP-7 primarily drives reparative rather than inflammatory pathways. Collectively, these results demonstrate that BMP-7 can favor the regeneration of cementoblast-like cells within the apical region after endodontic treatment, thereby contributing to a more functional and stable periapical healing process.

In vitro, BMP-7 stimulation activated SMAD-1/5/9 phosphorylation in human periodontal ligament fibroblasts (HPLFs), confirming engagement of the canonical BMP-SMAD signaling pathway. This activation pattern was inhibited by LDN-193189, a selective BMP type I receptor antagonist that suppresses receptor-mediated phosphorylation events [15,23]. These results provide direct mechanistic evidence that BMP-7 signaling is transmitted through the BMP receptor complex to SMAD-1/5/9, leading to the formation of the SMAD1/5/9-SMAD4 complex and subsequent transcriptional activation of downstream target genes involved in mineralized tissue differentiation. It is well established that RUNX2, a master transcription factor of osteo/cementogenic lineages, and BGLAP (osteocalcin), a late marker of mineralizing cells, are among these downstream targets. Therefore, the present findings support the conclusion that BMP-7 initiates intracellular signaling cascades in HPLFs that are analogous to those observed during cementoblast or osteoblast differentiation, even though fibroblasts are typically considered terminally differentiated cells. These data suggest that under certain conditions, fibroblasts in the periodontal ligament may retain plasticity that allows them to undergo cementoblast-like transformation when appropriately stimulated.

In the present study, BMP-7 enhanced the expression of cementoblast-associated markers, including SPON1, CEMP1, RUNX2, and BGLAP, particularly at 100 ng/mL, compared with the control group. These findings indicate that BMP-7 effectively promotes cementoblast-like differentiation of periodontal ligament fibroblasts. Consistent with this, BMP-7 treatment also increased alkaline phosphatase (ALP) activity and mineral deposition. A previous study demonstrated that HPLFs do not form calcified nodules even when cultured in osteogenic medium, underscoring the unique capacity of BMP-7 to induce mineralization under non-osteogenic conditions [24]. CEMP1, a key cementum-related protein, has been shown to promote cementoblastic differentiation while suppressing osteoblastic phenotypes, supporting its role as a specific marker of cementogenic lineage [25]. The ALP activity and marker expression were highest at 100 ng/mL BMP-7, whereas the 200 ng/mL group exhibited a diminished response, suggesting that an optimal concentration of BMP-7 may exist for the induction of cementoblast-like differentiation. Excessive BMP signaling is known to induce inhibitory molecules such as noggin or SMAD6/7, leading to receptor desensitization and negative feedback [9,26]. Therefore, an appropriate concentration range appears essential to maintain balanced BMP-7 activity and ensure effective differentiation. Furthermore, other markers specific to cementoblasts have been identified, including cementum attachment protein (CAP), β-tubulin III, and parathyroid hormone-related protein (PTHrP), which are involved in the structural and functional maturation of cementoblasts [27]. Although these markers were not examined in the present study, future investigations assessing their expression may help to further elucidate the detailed mechanisms underlying BMP-7–induced cementogenic differentiation.

Cementoblast differentiation is regulated by a network of intersecting pathways. Beyond BMP-SMAD, cross-talk with Sonic hedgehog and Wnt/β-catenin augments cementogenic output, and our system likely integrates such inputs [28,29]. Moreover, cell–cell communication also plays an important role. Direct adhesion between cementoblasts and PDL cells can induce cementoblast-like differentiation of the latter, suggesting juxtacrine reinforcement within the periodontal interface [30]. These lines of evidence provide a mechanistic context suggesting that while BMP-7 can initiate the process of cementoblast differentiation, it is unlikely to complete this program independently. This observation highlights the importance of considering combined approaches involving BMP-7 together with suitable scaffold materials or co-culture systems in future translational research.

The in vivo signal at the apex should also be interpreted in light of cell composition. Only a very small proportion of cells within the periodontal ligament exhibit stem or progenitor-like characteristics, whereas fibroblasts represent the predominant cell population [31]. Our choice to model HPLFs therefore reflects the most abundant and clinically relevant target. The fact that these fibroblasts activated BMP-SMAD signaling, increased SPON1/CEMP1/RUNX2/BGLAP expression, and deposited mineral suggests that resident ligament fibroblasts themselves can be recruited toward a cementoblast-like state by BMP-7 without the addition of exogenous stem cells. This aligns with the conceptual view that cementoblasts may be a distinct lineage adapted to the root-surface microenvironment, while also supporting the notion that PDL fibroblasts possess latent competence to adopt cementogenic features under appropriate signaling conditions [32].

From a translational perspective, recent preclinical work supports BMP-7 as a contributor to periodontal attachment gain and cementum deposition when delivered by cells or biomaterials [7,33]. Our data converge with that direction and, importantly, help bracket a working concentration range. Moving forward, it will be important to translate the mechanistic insights gained from this study into clinically applicable strategies for promoting cementum-mediated repair. Further work should address how BMP-7 can be delivered and regulated within the apical microenvironment to support long-term and biologically appropriate healing. Such efforts will provide a foundation for developing next-generation endodontic materials that harness biological signaling for tissue regeneration.

### Limitation of This Study

This study has certain limitations. First, the in vivo experiment used a split-mouth design without randomization, which may have introduced bias. Second, only one BMP-7 concentration was tested in vivo, limiting evaluation of concentration-response effects. Third, in vitro experiments were performed using a single human periodontal ligament fibroblast line, which may not fully represent biological variability. In addition, image analyses were performed by a single calibrated examiner to ensure consistency. However, the absence of independent verification or blinded cross-evaluation may have limited the assessment of observer bias. Future studies should include randomized designs, multiple concentrations, and donor-derived cell lines to validate these findings. Further preclinical and translational studies are warranted to confirm the safety, efficacy, and clinical feasibility of BMP-7-based strategies for endodontic and periodontal regeneration.

## 5. Conclusions

Within the limitations of this study, the findings suggest that BMP-7 promotes cementoblast-like differentiation of periodontal ligament fibroblasts through activation of the BMP-SMAD signaling pathway. Although no distinct cementum-like hard tissue was observed histologically, immunohistochemical analysis revealed increased Spon1 expression in the apical region, indicating that BMP-7 may stimulate cementogenic activity rather than bone-like repair. In vitro, BMP-7 upregulated key cementoblast-related markers and enhanced mineralization, supporting its role as a biological modulator within the cementogenic lineage. These results advance our understanding of BMP-7′s function in periodontal regeneration and provide a foundation for further studies aimed at optimizing its delivery, concentration, and safety for potential translational applications in regenerative endodontic therapy.

## Data Availability

The original contributions presented in this study are included in the article/supplementary material. Further inquiries can be directed to the corresponding author.

## Supplementary Materials

The following supporting information can be downloaded at https://www.mdpi.com/article/10.3390/dj13110494/s1, Table S1: Densitometric analysis of SMAD-1/5/9 and phospho-SMAD-1/5/9 Western blot bands. Table S2: Densitometric analysis of SPON1 and CEMP1 Western blot bands. Figure S1: Uncropped Western blot images showing SMAD-1/5/9, phospho-SMAD-1/5/9, and GAPDH. Figure S2: Uncropped Western blot images showing SPON1, CEMP1, and GAPDH.
