# Supplementary material for: Bone Morphogenetic Protein 7 Promotes the Differentiation of Periodontal Ligament Fibroblasts into F-Spondin-Expressing Cementoblast-like Cells During Root Canal Treatment—An In Vivo Rat Pulpectomy Model and In Vitro Human Fibroblast Study
Source: Dent J (Basel). 2025 Oct 25;13(11):494. doi: 10.3390/dj13110494 (PMC12651710; doi:10.3390/dj13110494)

Supplemental

Table 1

BMP-7 (100 ng/mL)	—	+	+	+	+	+
LDN-193189 (nM)	0	0	1	10	100	1000
pSMAD-1/5/9 / GAPDH	1	1.5218	1.0621	0.7041	0.6253	0.2791
SMAD-1/5/9 / GAPDH	1	1.0981	1.0277	0.8582	0.8906	0.7520

Table 2

BMP-7 (ng/mL)	0	100	200
SPON1 / GAPDH	1	4.9317	3.5138
CEMP1 / GAPDH	1	46.7918	27.4654

Supplemental

Figure 6

GAPDH

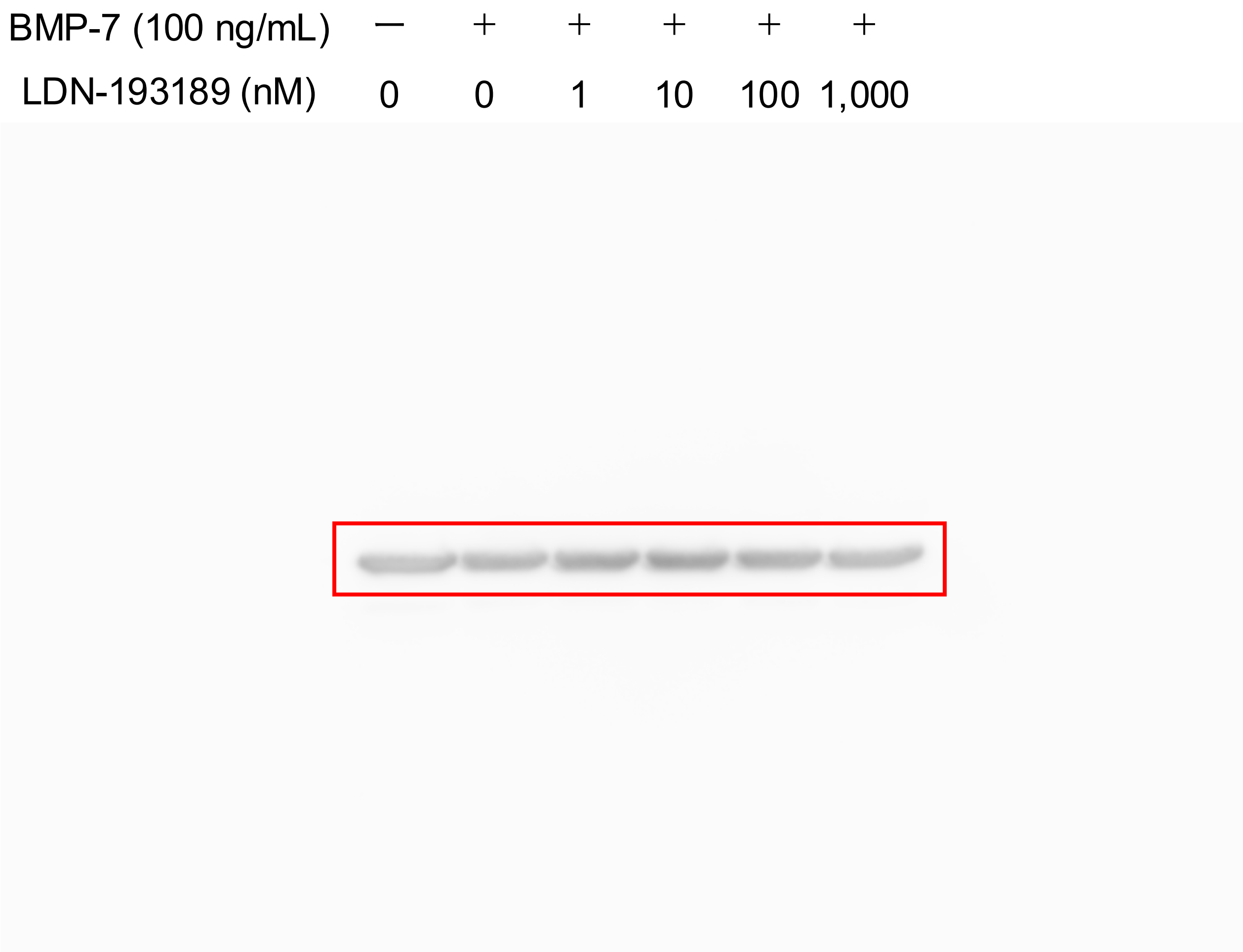

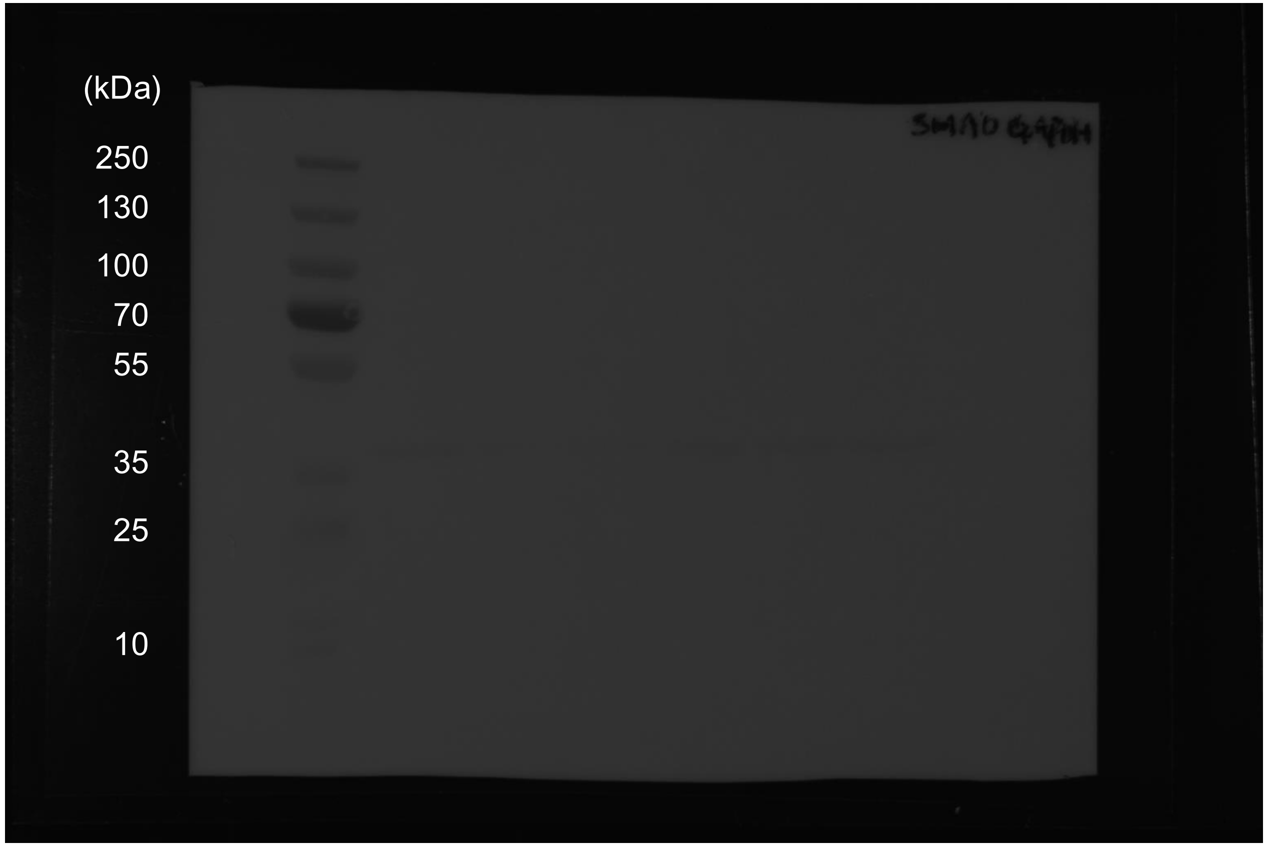

Supplemental

Figure 6

pSMAD-1/5/9

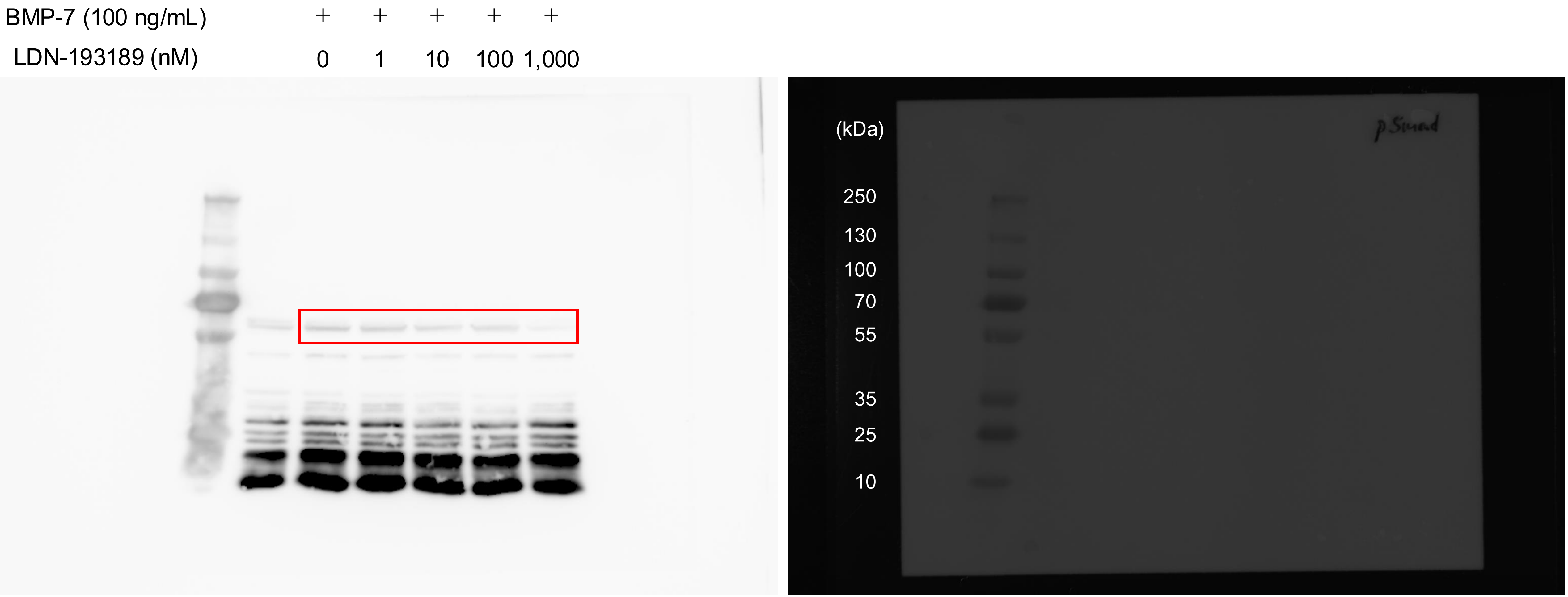

Supplemental

Figure 6 pSMAD-1/5/9

BMP-7 (100 ng/mL) –
LDN-193189 (nM) 0

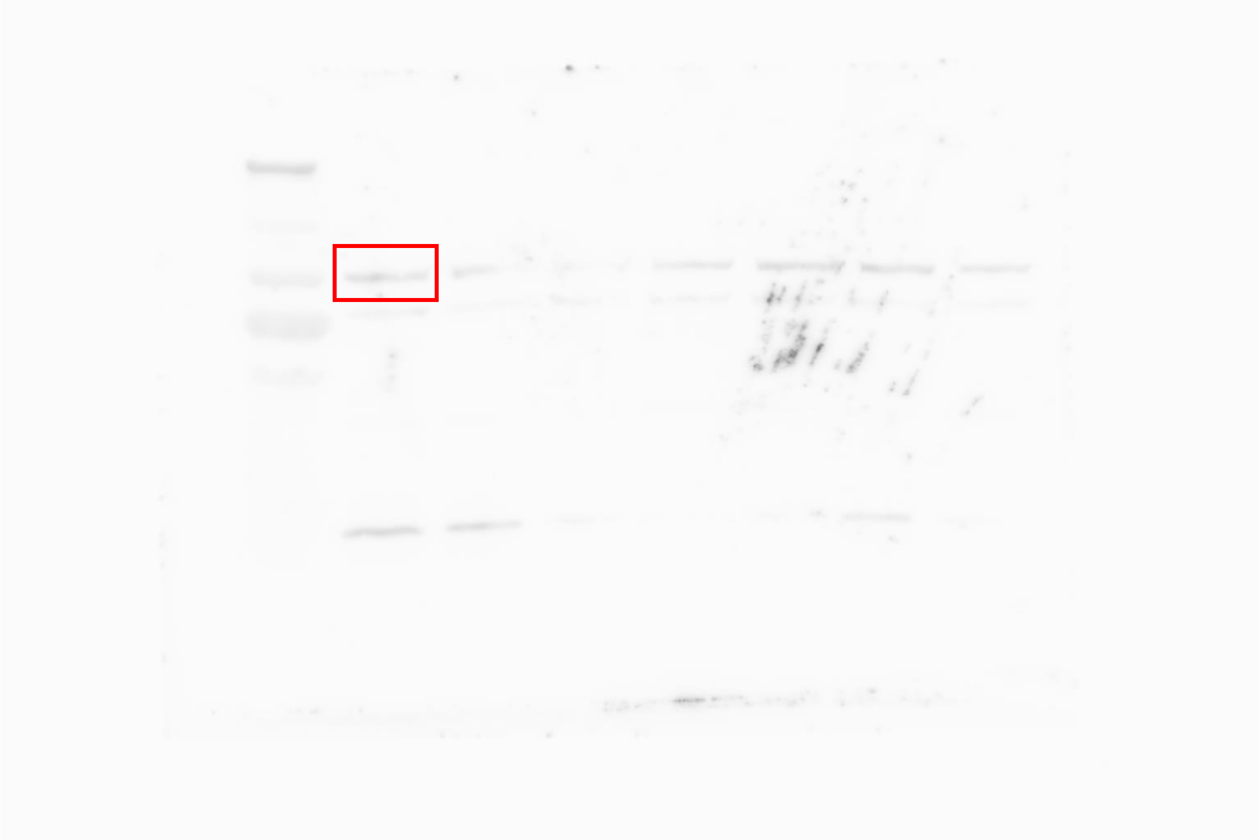

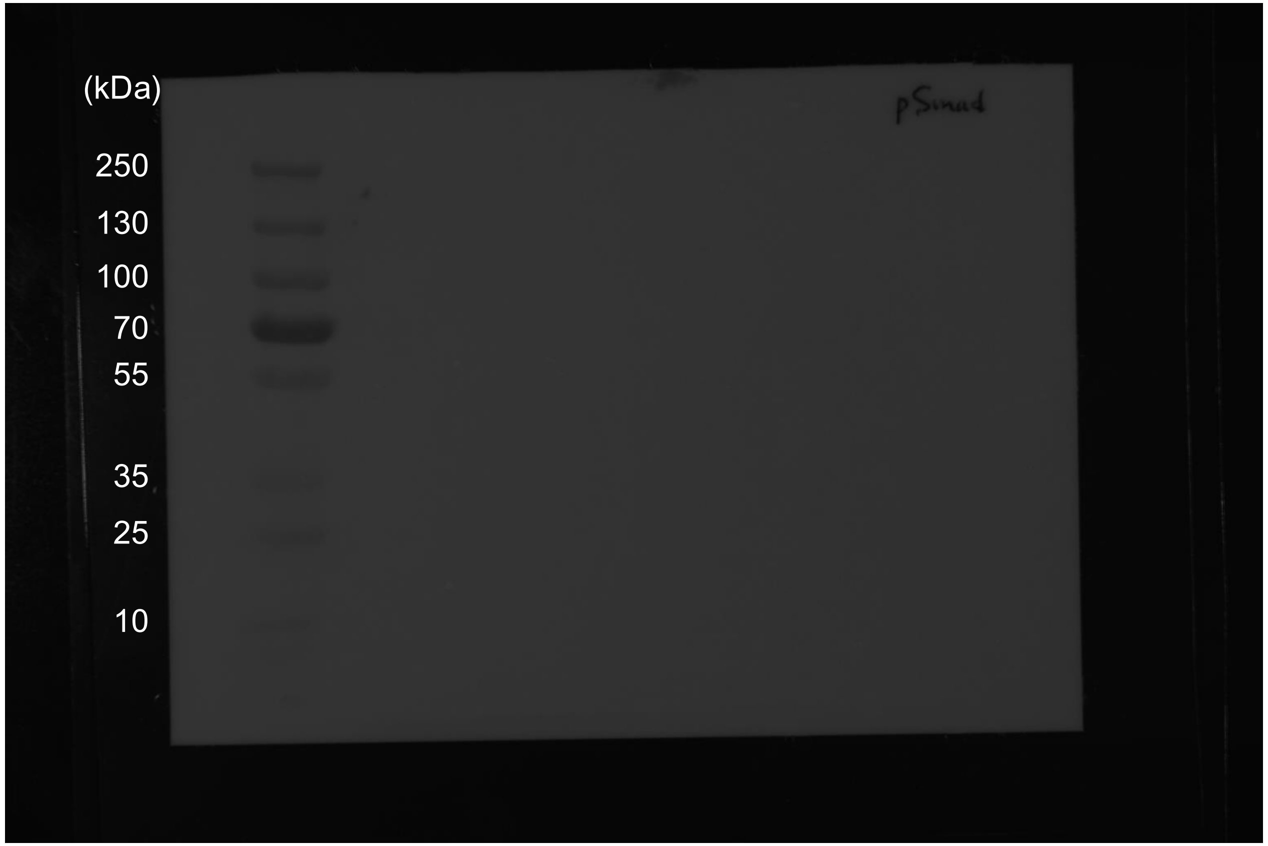

Supplemental

Figure 6

SMAD-1/5/9

BMP-7 (100 ng/mL)	—	+	+	+	+	+
LDN-193189 (nM)	0	0	1	10	100	1,000

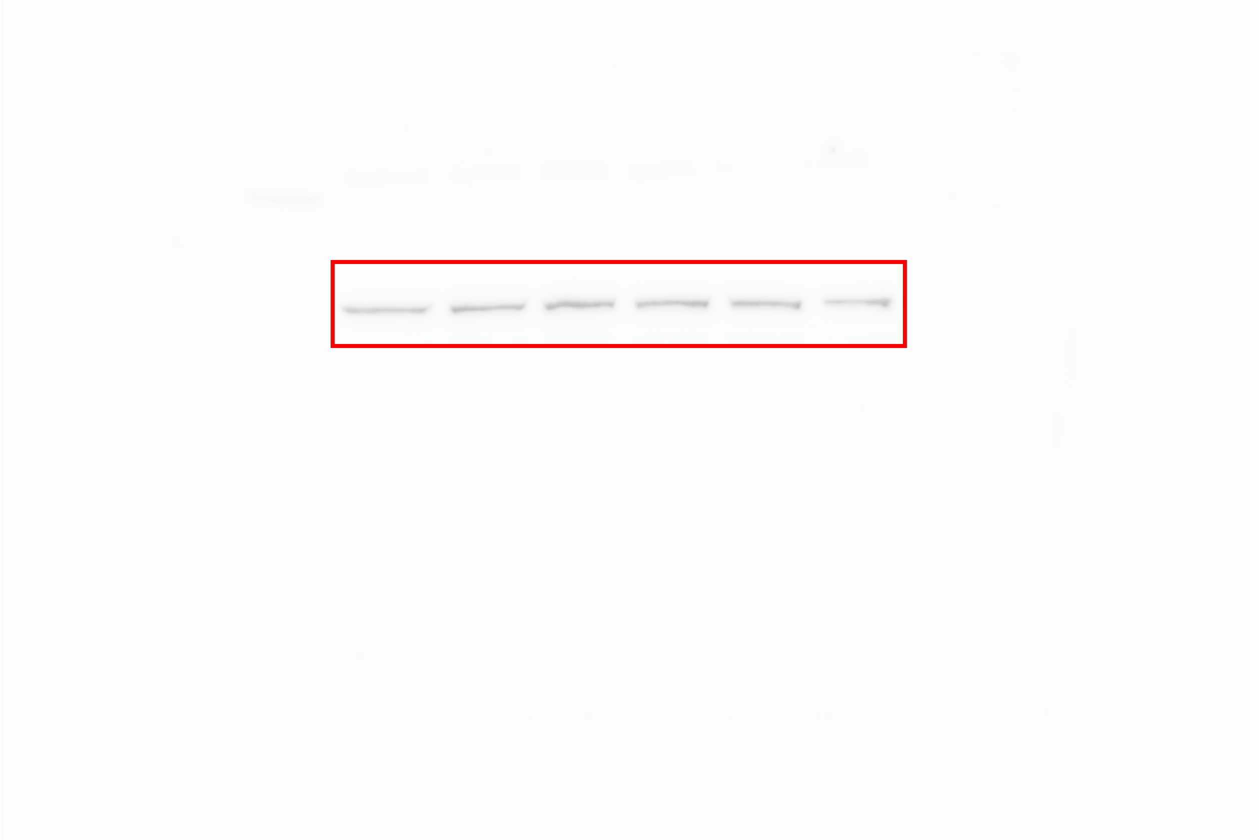

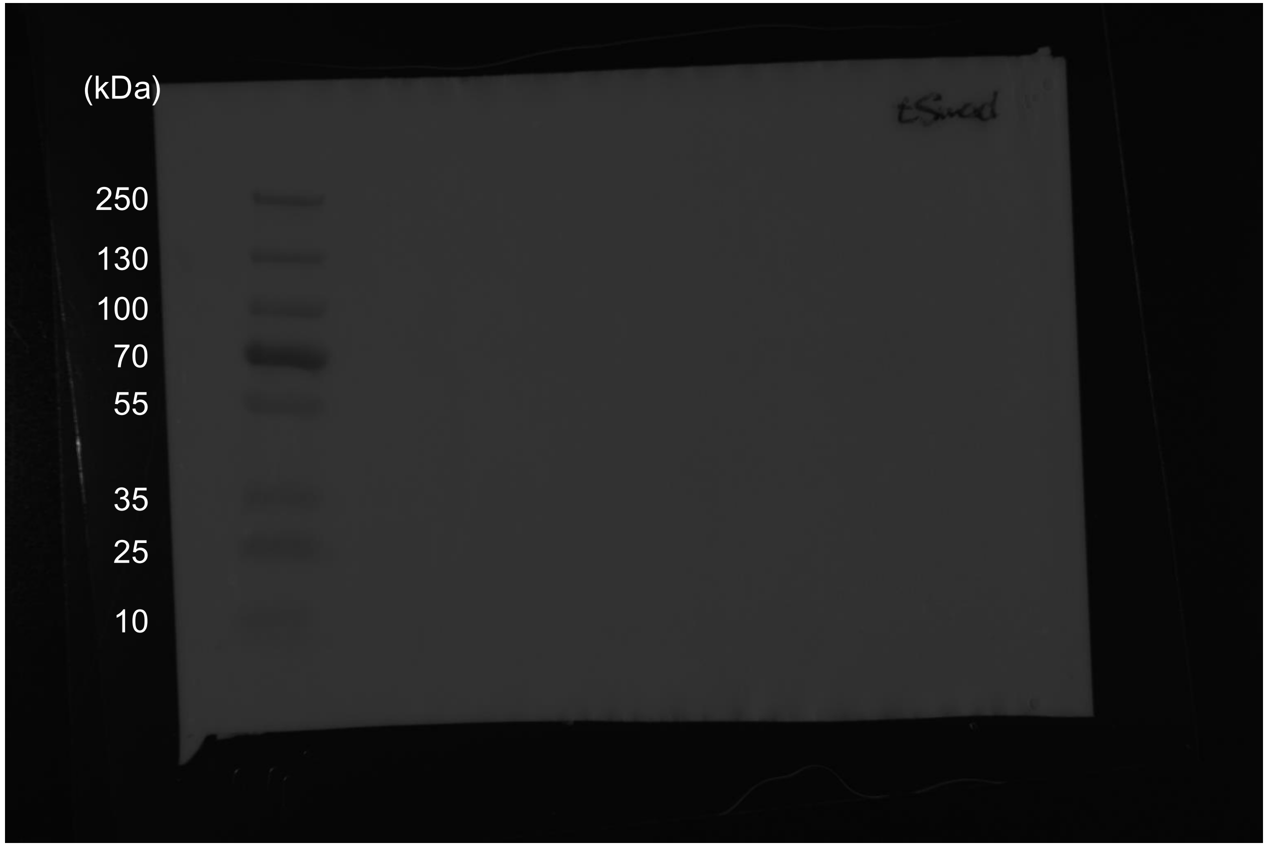

Supplemental

Figure 8

GAPDH

BMP-7 (ng/mL) 0 100 200

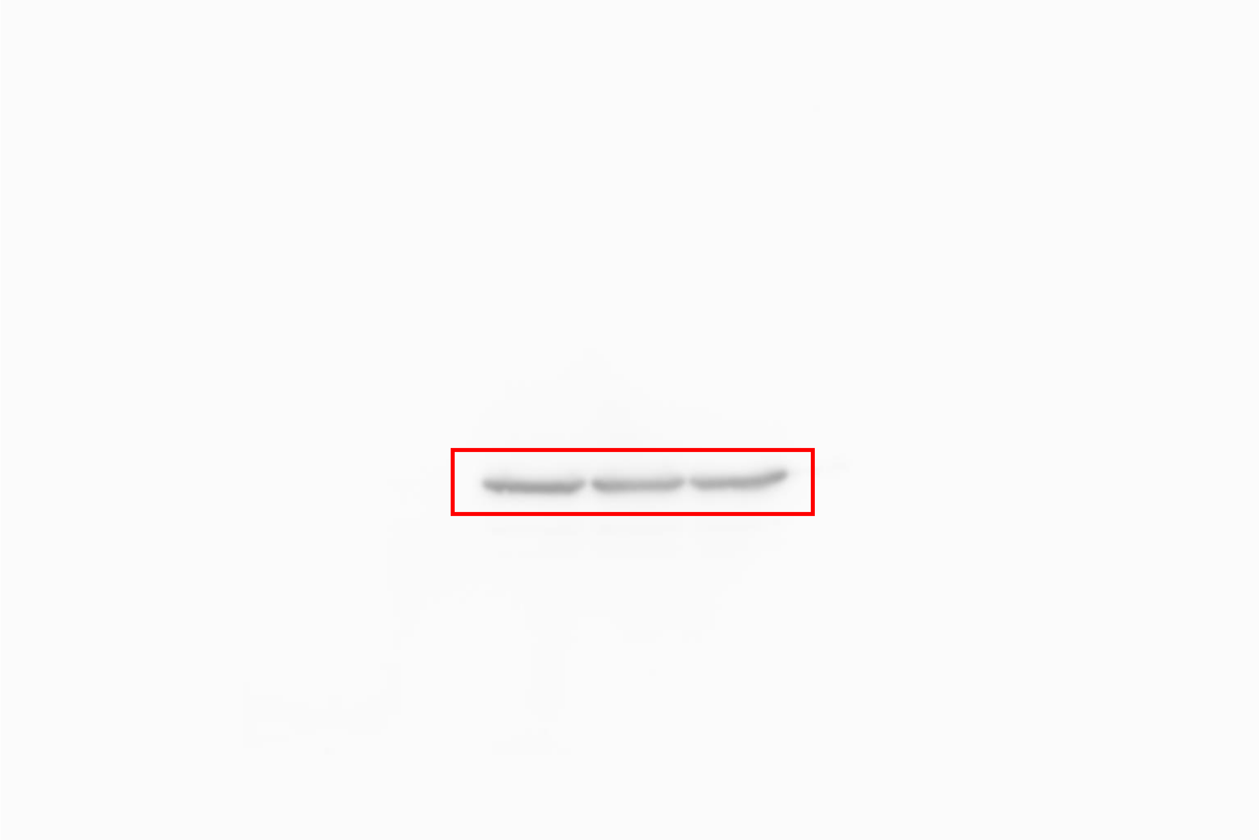

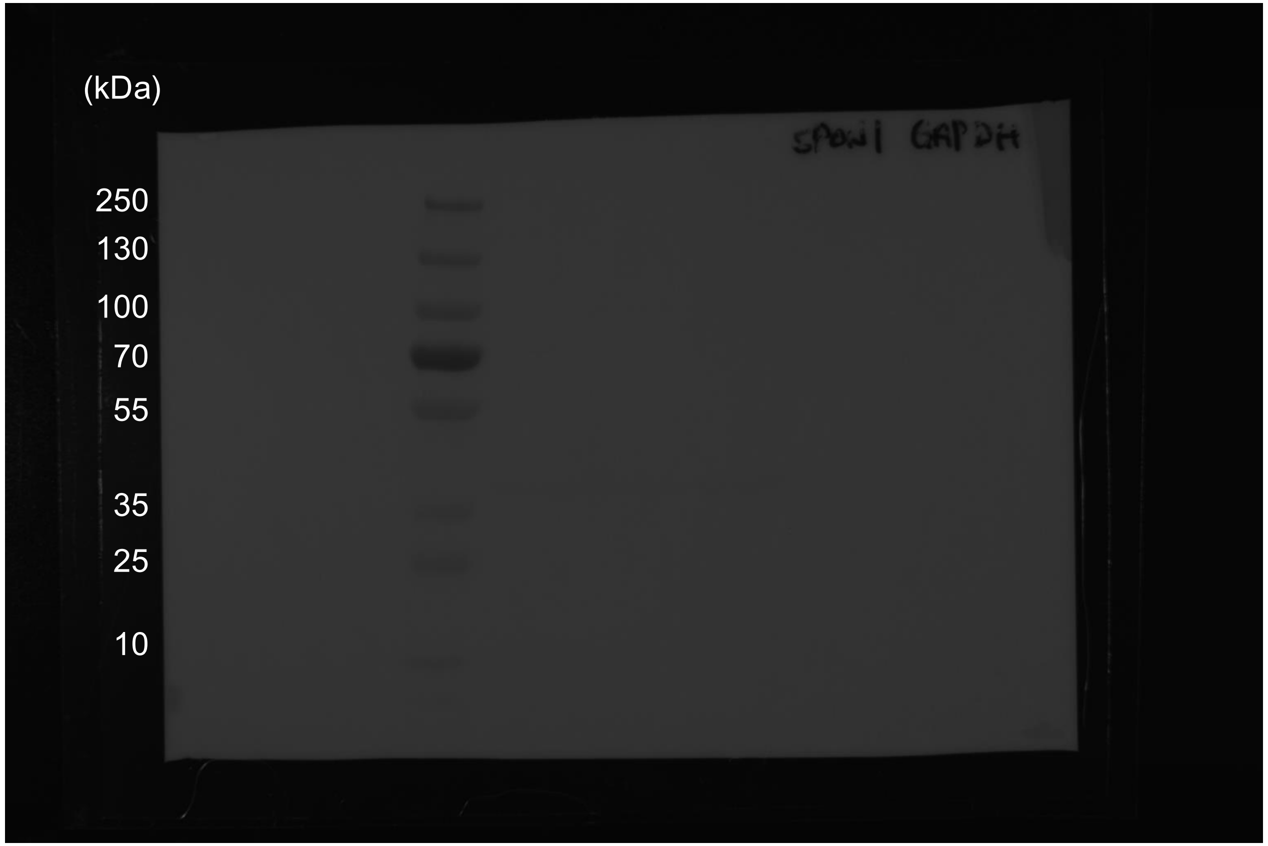

Supplemental

Figure 8

SPON1

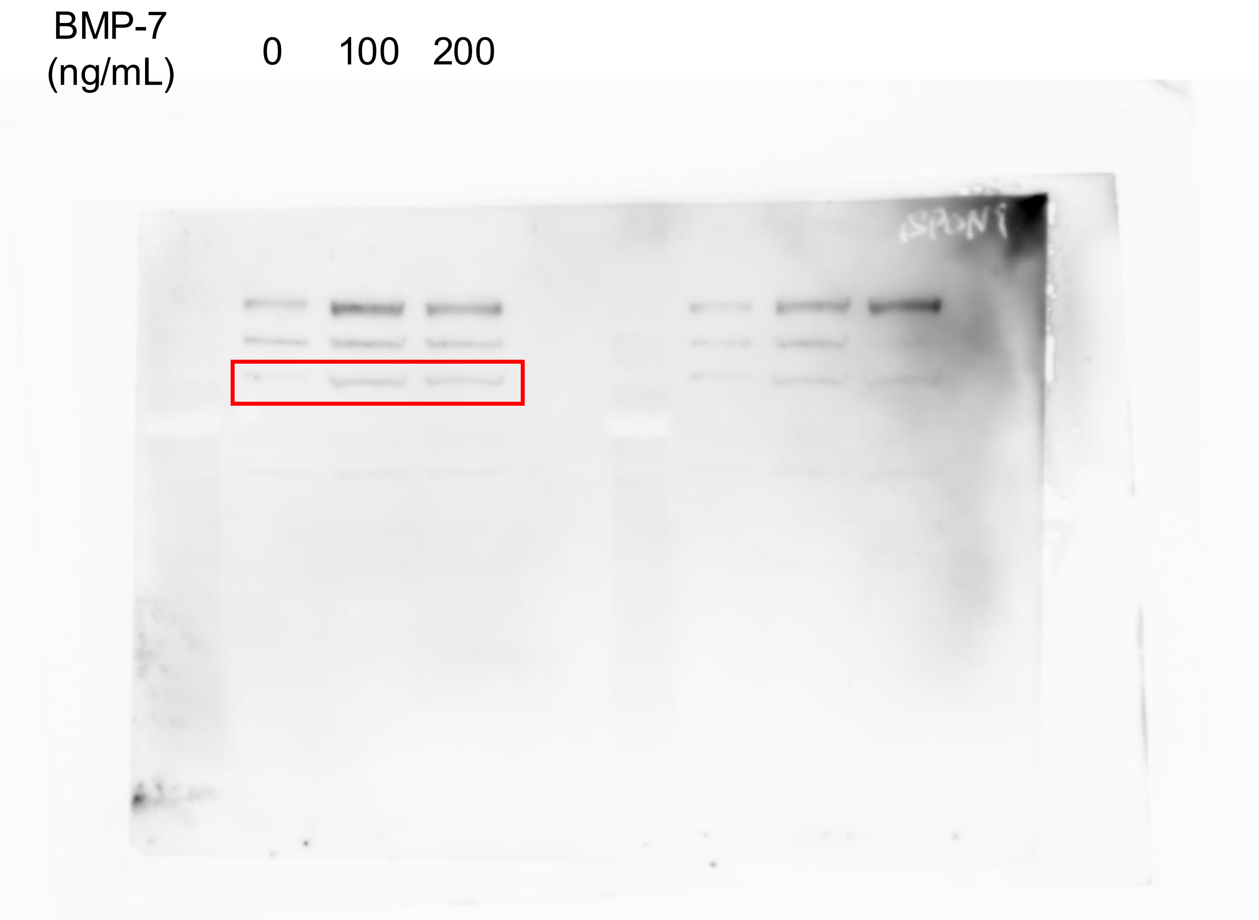

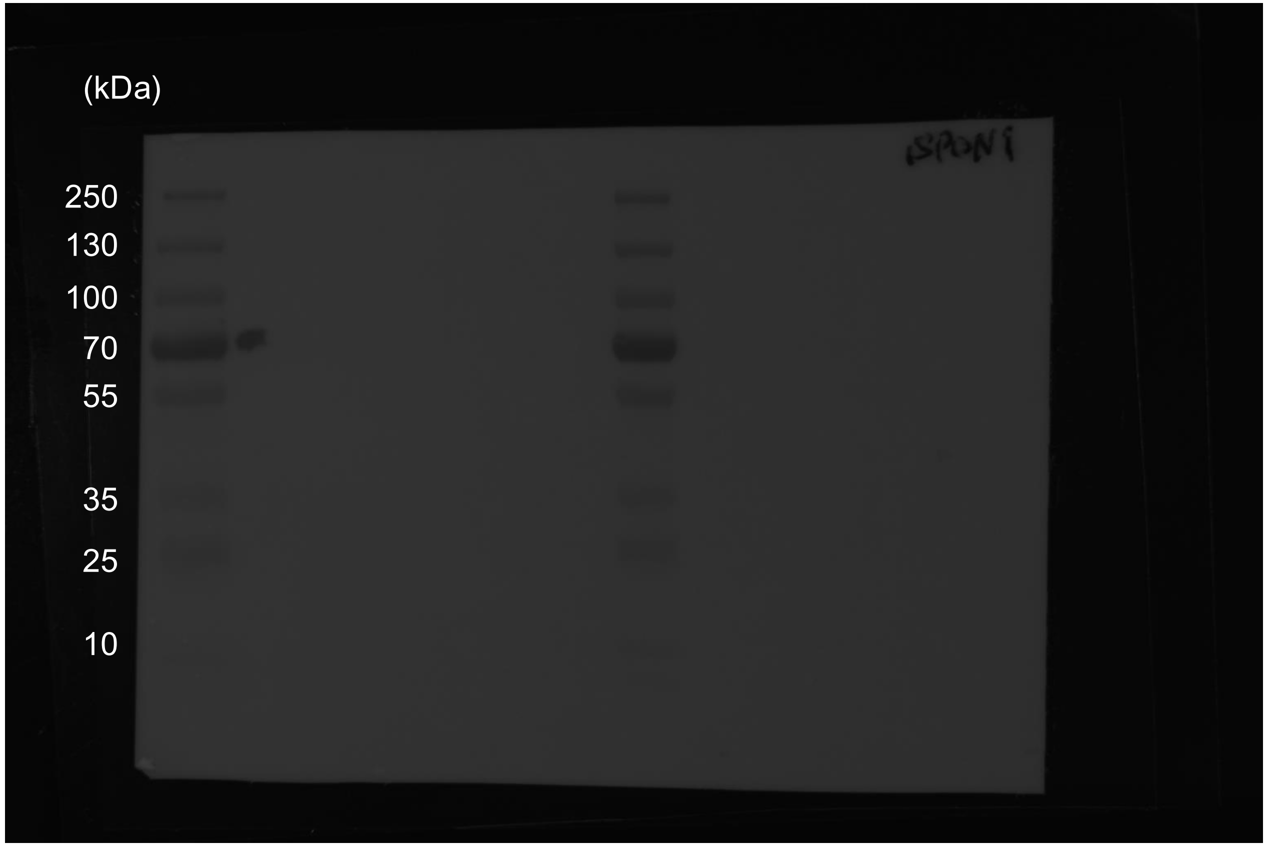

Supplemental

Figure 8

CEMP1

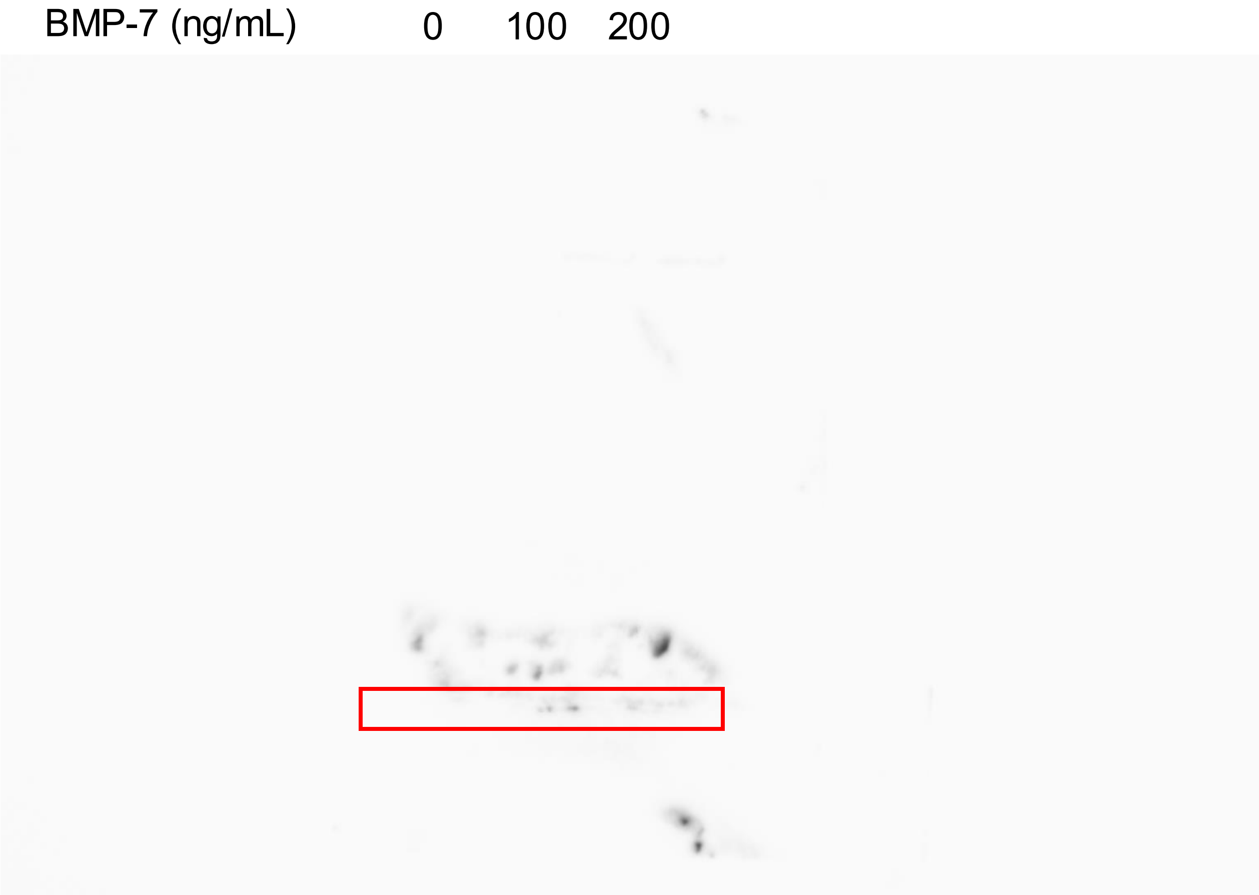

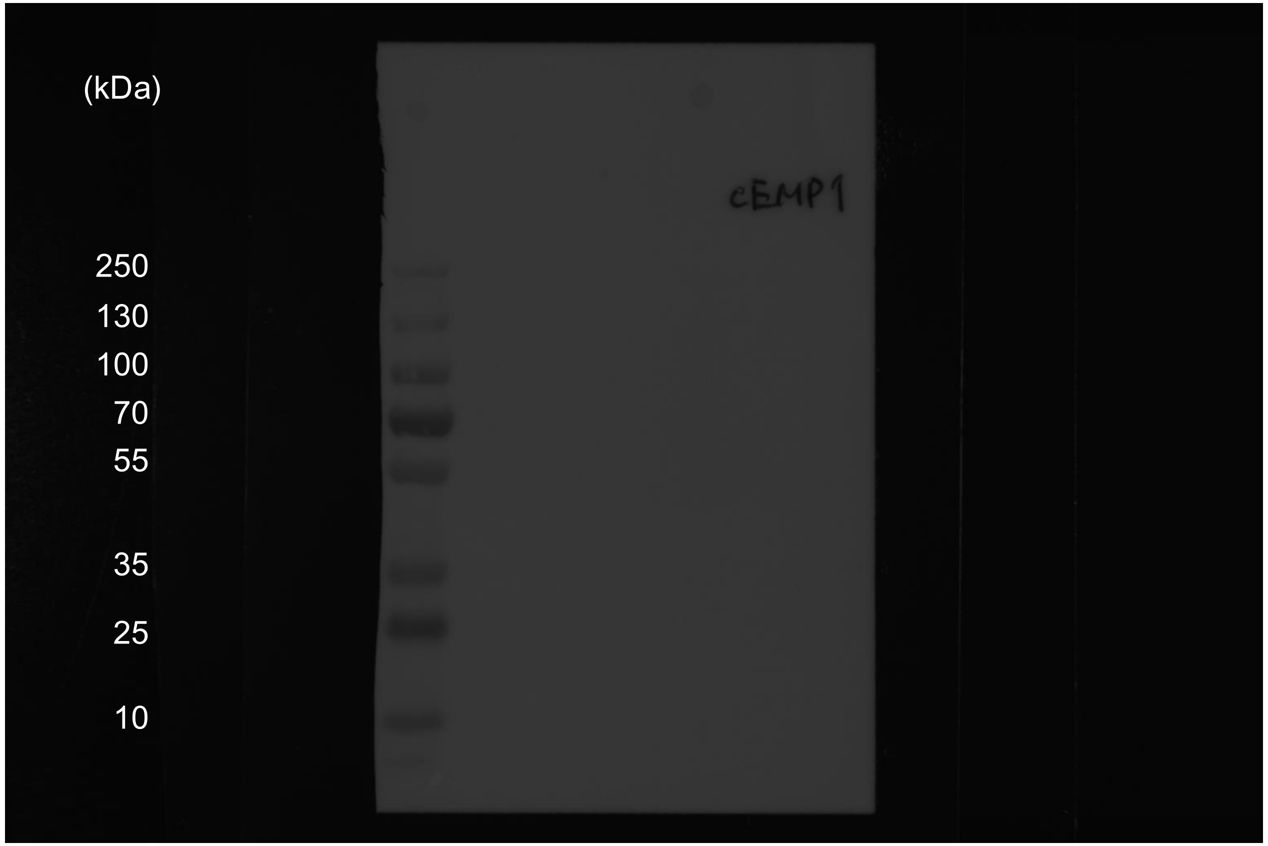

Supplement: Supplementary file 1 [file dentistry-13-00494-s001.zip › dentistry-3831329-supplementary.pdf]
